# Lithium neurotoxicity – a case report and review of the literature

**DOI:** 10.1192/j.eurpsy.2023.2136

**Published:** 2023-07-19

**Authors:** C. Almeida Rodrigues, A. Carvalho, F. Martins Costa, V. Silva de Melo

**Affiliations:** 1 Psychiatry, Centro Hospitalar do Médio Tejo, Tomar; 2 Neurology, Centro Hospitalar de Vila Nova de Gaia e Espinho, Vila Nova de Gaia, Portugal

## Abstract

**Introduction:**

Lithium, a mood stabilizer, is a commonly prescribed and effective treatment for bipolar affective disorder. It´s excreted almost exclusively by the kidneys with a half-life primarily determined by renal function. Chronic intoxication results from an insidious accumulation of lithium in a chronically medicated patient (due to a reduction in renal function secondary to volume depletion, a new medication, et cetera). Patients often present with neurologic findings, including tremor, ataxia, dysarthria, confusion and neuromuscular excitability.

**Objectives:**

The objective of this report is to describe a clinical case of lithium neurotoxicity (myoclonus and encephalopathy), along with a review of the literature on the topic.

**Methods:**

We describe a case of lithium neurotoxicity, along with a brief non-systematic review of the literature on lithium toxicity. We conducted a PubMed bibliographic search using keywords such as “lithium intoxication”, “lithium neurotoxicity”, “lithium encephalopathy” and “lithium intoxication treatment”.

**Results:**

A women aged 81 was brought to the emergency department by her daughter following 1 week of asthenia, diarrhoea, periods of confused speech and involuntary movements. In the previous week, the patient had been diagnosed with COVID-19. Her past medical history is significant for bipolar affective disorder, hypertension, diabetes mellitus, dyslipidemia and asthma. The patient has been treated with following drugs: lithium carbonate (no recent change of dose and previous serum levels around 1mmol/L), quetiapine, lisinopril, metformin, simvastatin, formoterol and budesonide. On the first examination, she had an exuberant multifocal myoclonus. Posteriorly, she became somnolent, with language impairment (verbal perseveration, echolalia) and dysarthria. Investigations revealed renal impairment (creatinine 1,5 mg/dL, blood urea nitrogen 42 mg/dL) and supratherapeutic lithium levels (lithium serum level 1,7 mmol/L). Computed tomography scan of the brain was negative for acute injuries. The electroencephalogram showed triphasic waves (1-1,5 Hz). Encephalopathy secondary to lithium intoxication was diagnosed (probably in the context of acute kidney injury precipitated by hypovolaemia – diarrhoea). Lithium was stopped and intravenous isotonic fluids were given. After 1 week, her myoclonus resolved and over the following week the other signs resolved as well. The patient was later discharged to her daughter’s home, with follow-up neurology and psychiatry visits.

**Conclusions:**

Both reversible and irreversible neurotoxicity related to lithium have been reported, specially occurring alongside chronic intoxication. If not addressed, impaired consciousness can lead to coma and death. A high clinical suspicion is needed for prompt diagnosis and treatment (intravenous fluids and sometimes haemodialysis are warranted).

**Disclosure of Interest:**

None Declared

